# Transport and fate of human expiratory droplets—A modeling approach

**DOI:** 10.1063/5.0021280

**Published:** 2020-08-01

**Authors:** Binbin Wang, Huijie Wu, Xiu-Feng Wan

**Affiliations:** 1 Department of Civil and Environmental Engineering, University of Missouri, Columbia, Missouri 65211, USA; 2 MU Extension, University of Missouri, Columbia, Missouri 65211, USA; 3 Missouri University Center for Research on Influenza Systems Biology (CRISB), University of Missouri, Columbia, Missouri 65211, USA; 4 Department of Molecular Microbiology and Immunology, University of Missouri, Columbia, Missouri 65211, USA; 5 Department of Electrical Engineering and Computer Science, University of Missouri, Columbia, Missouri 65211, USA; 6 Bond Life Sciences Center, University of Missouri, Columbia, Missouri 65211, USA

## Abstract

The transport and fate of human expiratory droplets play a key role in the transmission of respiratory infectious diseases. In this paper, we present a modeling approach to understand the fundamental dynamics of exhaled droplets in human respiratory activities. The model solves a series of governing equations of droplets and uses a continuous random walk model to simulate turbulent fluctuations in violent expiratory events. The validation of the model shows the improvement in the prediction of dispersion of median-sized droplets. We show that these droplets are sensitive to environmental conditions, including temperature, humidity, and ambient flows. Applying the model to a set of idealized conditions such as free-fall and continuous jets, we demonstrate significantly different impacts of environmental parameters on droplets with different sizes. Using a realistic droplet size distribution and cough duration, we quantify the transport and fate of droplets in the near field of source and the potential influences by ambient conditions. The model we developed from this study could be applied to study the mechanisms for airborne pathogens, e.g., influenza virus and new coronavirus.

## I. INTRODUCTION

The ongoing Coronavirus Disease 2019 (COVID-19) pandemic caused by a novel coronavirus known as SARS-CoV-2 iterates an important question about how viruses are transmitted through natural respiratory activities such as breath, talk, speech, cough, and sneeze (e.g., Mittal *et al.*, 2020; Bourouiba, 2020; and Asadi *et al.*, 2020). The dynamics of virus transmission is not well understood, with one challenge being the complicated fluid and flow characteristics involved in the fate and transport of virus, including source dynamics (e.g., exhale velocity and temperature, droplet sizes, virus load, and droplet–virus correlations), ambient conditions (e.g., mean and turbulent flows, temperature, and humidity), and virus dynamics (e.g., virus viability and infectious rate) (e.g., Lindsley *et al.*, 2015; Feng *et al.*, 2020; Dbouk and Drikakis, 2020a; and Mittal *et al.*, 2020). Understanding the fundamental fluid dynamics of expiratory virus-laden droplets is critical to the prediction of the transport and fate of droplets and associated potential threats of infectious disease transmission and will provide quantitative guidance for making a public health policy for disease mitigation, e.g., decisions on social distancing and face covering in various indoor and outdoor environments (Dbouk and Drikakis, 2020b; Verma *et al.*, 2020).

Human respiratory activities produce droplets with a wide range of sizes, depending on the type of activities and physical and health conditions of individuals. The typical sizes of human expiratory droplets are in the range of 10^−1^ *μ*m–10^3^ *μ*m (e.g., Duguid, 1945; Yang *et al.*, 2007; Chao *et al.*, 2009; Xie *et al.*, 2009; Han *et al.*, 2013; and Asadi *et al.*, 2019). This wide range of droplet sizes results in various flow-following capabilities and falling speeds in the respiratory flows. For instance, a 10 *μ*m droplet has the terminal velocity of 3 mm/s, whereas a 1000 *μ*m droplet has the terminal velocity of 3.86 m/s. In the meantime, the particle relaxation time of a 10 *μ*m and a 1000 *μ*m droplet is 0.0003 s and 0.39 s, respectively. Given their sizes, the Stokes number for 1000 *μ*m droplets is one order of magnitude higher than that of 10 *μ*m droplets in the same air velocity. Hence, the air flow has much weaker impact on the 1000 *μ*m droplet (i.e., stronger inertia effect) than the 10 *μ*m droplet. Because the human expiratory droplets span a wide size range even in a single event, the transport of these droplets has significant temporal and spatial variability in air flows from the perspective of fluid kinematics, let alone various time scales of the concurrent thermodynamic processes.

Respiratory infectious illness can be transmitted by viruses that are passed along from hosts to recipients through the dispersal of virus-laden droplets in the normal respiratory activities (Milton *et al.*, 2013; Gralton *et al.*, 2013; and Zhou *et al.*, 2018). Violent expiratory events such as coughing and sneezing have high initial velocity (∼10 m/s) and can spread droplets within 2 m–3 m (Bourouiba *et al.*, 2014), which can be extended up to 7 m–8 m due to strong turbulence and buoyant effects (Bourouiba, 2020). Many studies have reported viable viruses in human respiratory activities and virus detection in air samples away from patients (e.g., Bischoff *et al.*, 2013; Lindsley *et al.*, 2012; and 2015). Despite no well-constrained dynamics of airborne virus transmission, it is apparent that the transport of droplets through human respiratory activities plays a key role in the transmission of respiratory infectious diseases.

Exhaled droplets are transported away from the source (i.e., nose and mouth) in respiratory activities, following flow patterns with the expiratory event controlled time and velocity scales and their interaction with ambient air flows. Human cough generates a maximal initial velocity on the scale of ∼10 m/s (Chao *et al.*, 2009; Tang *et al.*, 2008; VanSciver *et al.*, 2011; Kwon *et al.*, 2012; and Zhu *et al.*, 2006) within a typical time scale of 0.5 s (Gupta *et al.*, 2009). Yang *et al.* (2007) found a positive correlation between cough flow rate and droplet population. These properties of respiratory flows and associated droplets provide necessary information to determine the evolving droplet dynamics within a rapid physical process in the near field of the source before the end of the respiratory activities. The droplet dynamics in the near field determines where, when, and which droplets would settle on the solid surface (e.g., ground and table) as a potential infectious source for fomite transmission (Lindsley *et al.*, 2010; Wei and Li, 2016). This near field dynamics also serves as the initial condition and can be coupled with far field transport by ambient air flows and turbulence (e.g., Wei and Li, 2016; Zhu *et al.*, 2006; Thatiparti *et al.*, 2017; and Dbouk and Drikakis, 2020a).

Both droplet properties and flow characteristics play a key role in the transport of droplets and transmission of associated infectious diseases. Xie *et al.* (2007) demonstrates the evaporation time and settling time as a function of droplet diameter, known as the Wells-curve (Wells, 1934). They added an additional curve that illustrates the droplets escaping from cough jets. In their analyses, the influence of turbulence was not included, and hence, the conclusion is limited for practical purposes. Wei and Li (2015) applied a discrete random walk (DRW) model to simulate the turbulent transport of droplets in a cough jet, providing better predictions of droplet transports in the lateral direction of the cough jet. A critical information from the model result is that the median-sized droplets (30 *μ*m–50 *μ*m in their simulation) are sensitive to ambient humidity. When ambient air is dry, the median-sized droplets can shrink to adequately small sizes within the cough jet and would not escape the jet. In humid air, the suppressed shrinkage of droplet sizes due to suppressed evaporation can maintain adequate inertia so that these droplets are able to leave the cough jet. One limitation of their model is that DRW is not a good representation of the continuously correlated turbulent fluctuations as it uses a discrete random walk algorithm. The random process is only imposed when the time scale is larger than the particle–flow interaction time scale, defined based on the particle relaxation time and eddy lifetime. Wei and Li (2015) acknowledged this limitation and pointed out that their model does not perform well for median-weighted particles (87 *μ*m corn pollen in their validation). Note that the corn pollen has approximately the same relaxation time as the expiratory droplets with the same sizes. Hence, the limitation in predicting median-weighted particles may induce considerable errors in predicting the transport of virus-laden droplets in human respiratory flows.

To address the above shortcomings in predicting human respiratory flows and associated transport of droplets, here, we present a droplet transport and fate model to improve the prediction of spreading of median-sized droplets, which are sensitive to ambient environment and critical to the decision of public health responses. In our model, we use a continuous random walk (CRW) algorithm, an approach designed to simulate the continuous and auto-correlated nature of turbulent fluctuations (Bocksell and Loth, 2001; Mofakham and Ahmadi, 2019; and 2020) and mathematically represented by a stochastic differential equation (SDE) and its numerical form. Section II presents the governing equations that derive the aerodynamic and thermodynamic processes of the particles in the model. The CRW model and the equations of the cough jet are also presented in Sec. II. The validation of the model is given in Sec. III, demonstrating the model skills in predicting both thermodynamics and kinematics. In Sec. IV, with various applications of the model, we illustrate the behavior of the human expiratory droplets in a set of idealized scenarios in indoor environments. In Sec. V, we discuss limitations of the model and caveats to the presented study. The concluding remarks and a brief outlook for future research are summarized in Sec. VI.

## II. METHODS

### A. Lagrangian particle model

The Lagrangian particle model (LPM) computes the aerodynamic and thermodynamic properties of individual particles, where the particles are predefined by their type and properties (i.e., density, size, temperature, and hu). The location (*x*_*i*_, *i* = 1, 2, 3, where indices 1 and 2 represent the horizontal directions and 3 represents the vertical direction in the Cartesian coordinate system) and velocity (*u*_*p*,*i*_) of each particle are calculated along its path based on the governing equation (Wei and Li, 2015; Chao and Wan, 2006),

$$\frac{dx_{i}}{dt}=u_{p,i},$$ (1)

$$\frac{du_{p,i}}{dt}=\frac{3\rho_{g}C_{d}}{4d_{p}\rho_{p}}\left(u_{i}-u_{p,i}\right)|u_{i}-u_{p,i}|+g_{i},$$ (2)

where *ρ*_*p*_ and *ρ* are the density of the particle and ambient air, respectively, *d*_*p*_ is the particle diameter, *C*_*d*_ is the drag coefficient, *u*_*i*_ is the instantaneous air velocity that consists of a mean and a turbulent component, and *g*_3_ = −9.81 m/s^2^ is the gravitational acceleration. The drag coefficient *C*_*d*_ is calculated (Wei and Li, 2015; Goossens, 2019),

$$C_{d}=\left\{\begin{array}{cc} \frac{24}{Re},\; & Re\leq 1\\ \frac{24}{Re}\left(1+0.15Re^{0.687}\right),\; & 1<Re\leq 1000\\ 0.44,\; & Re>1000, \end{array}\right.$$ (3)

where the Reynolds number is defined as *Re* = *d*_*p*_|*u*_*i*_ − *u*_*p*,*i*_|/*ν* with *ν* being the kinematic viscosity of air.

In respiratory flows, the mass and temperature of each droplet are calculated according to mass and heat transfer equations, following a series of developments in droplet evaporation and condensation processes (Kukkonen *et al.*, 1989; Walton, 2004). Mass transfer occurs at the droplet surface due to the diffusive flux of vapor (*I*_v_ = −*dm*_*p*_/*dt*),

$$\frac{dm_{p}}{dt}=\frac{2\pi d_{p}pM_{p}C_{T}D_{\infty }Sh}{RT_{\infty }}\ln\left(\frac{1-p_{v,p}/p}{1-p_{v,\infty }/p}\right),$$ (4)

where *M*_*p*_ is the molecular weight of the droplet, *p* is the total pressure, *D*_∞_ is the ambient binary diffusion coefficient, *R* is the universal gas constant, *T*_∞_ is the ambient temperature with unit of Kelvin, and *p*_v,*p*_ and *p*_v,∞_ are the vapor pressure at the droplet surface and in the ambient, respectively. The factor *C*_*T*_ accounts for the temperature dependence of the diffusion coefficient, defined as

$$C_{T}=\frac{T_{\infty }-T_{p}}{T_{\infty }^{\lambda -1}}\frac{2-\lambda }{T_{\infty }^{2-\lambda }-T_{p}^{2-\lambda }},$$ (5)

with a coefficient *λ* specified for droplets (Kukkonen *et al.*, 1989).

In Eq. (4), the Sherwood number accounts for enhanced mass transfer in the turbulent boundary layer of the droplet surface, calculated by *Sh* = 1 + 0.3*Re*^1/2^*Sc*^1/3^ with Schmidt number *Sc* = *ν*/*D*_*∞*_. When the droplet is at rest, *Sh* = 1 and mass transfer is entirely controlled by the molecular binary diffusion process.

The temperature of the droplet is calculated (Wei and Li, 2015),

$$c_{p}m_{p}\frac{dT_{p}}{dt}=2\pi d_{p}K_{g}\left(T_{\infty }-T_{p}\right)Nu-L_{v}I_{v}-\pi d_{p}^{2}\Gamma \left(T_{p}^{4}-T_{\infty }^{4}\right),$$ (6)

where *c*_*p*_ is the specific heat capacity of the droplet, *K*_*g*_ is the thermal conductivity of air, *L*_v_ is the latent heat of droplet vapor, and Γ is the Stefan–Boltzmann constant.

The three terms on the right-hand side in Eq. (6) are thermal conduction, evaporation, and thermal radiation. Similar to mass transfer, enhanced thermal conduction in turbulent flow should be considered, given by the factor of Nusselt number *Nu* = 1 + 0.3*Re*^1/2^*Pr*^1/3^ with Prandtl number *Pr* = *cμ*/*K*_*g*_ (*c* and *μ* are the specific heat and dynamic viscosity of ambient air).

Droplets have various compositions (e.g., salt, protein, and surfactant), which vary for different individuals, their health conditions, and the respiratory activities (i.e., breath, talk, cough, and sneeze) (Vejerano and Marr, 2018). A simplified treatment is to decompose the droplet to liquid and solid phases, where the solid phase is contributed by NaCl (Xie *et al.*, 2007; Wei and Li, 2015). Hence, the *c*_*p*_*m*_*p*_ in Eq. (6) can be written as *c*_*l*_*m*_*l*_ + *c*_*s*_*m*_*s*_, where the subscripts *l* and *s* represent the liquid and solid phases, respectively.

We follow Wei and Li (2015) to include the effect of the surface curvature of the droplet and the solid phase on the vapor pressure of the droplet, namely, Kelvin effect. This correction considers the concentration of NaCl and its influence on the final nucleus sizes when the droplets lose all of the liquid content.

When the air flow field is given, i.e., instantaneous air velocity *u*_*i*_ at any location is known, the ordinary differential equation (ODE) system [Eqs. (1), (2), (4), and (6)] can be solved to track the location, velocity, mass (used to calculate the droplet diameter), and temperature of each droplet. In almost any natural and indoor environments, turbulence exists and helps spread particles in air flow, which needs additional treatment.

### B. Modeling of turbulent fluctuation

We apply a continuous random walk (CRW) model to simulate the turbulent fluctuating velocity in air. A stochastic differential equation (SDE) is used to simulate instantaneous air fluctuating velocity, known as the Langevin equation,

$$\frac{du_{i}^{\prime }}{dt}=-\alpha u_{i}^{\prime }+\beta \xi_{i},$$ (7)

where $u_{i}^{\prime }$ is the fluctuating velocity component of air, *ξ*_*i*_ is a Gaussian white noise, and *α* and *β* are coefficients that determine the stochastic nature of the turbulent process. The magnitude of *α* represents the autocorrelation of flow fluctuation, and the magnitude of *β* represents the relative importance of the random process with respect to the velocity correlations. Legg and Raupach (1982) showed *α* = 1/*τ*_*i*_ and $\beta =\sigma_{i}{\left(2/\tau_{i}\right)}^{0.5}$, with *τ*_*i*_ being the Lagrangian time scale and *σ*_*i*_ being the root-mean-square (rms) of velocity fluctuations at each direction.

A discrete format of Eq. (7) is equivalent to the Markov chain equation, which has been used in Bocksell and Loth (2001) for CRW modeling,

$$u_{i}^{\prime }\left(t+\Delta t\right)=u_{i}^{\prime }\left(t\right)\exp\left(-\Delta t/\tau_{i}\right)+\sigma_{i}{\left(1-\exp\left(-2\Delta t/\tau_{i}\right)\right)}^{1/2}\xi_{i}.$$ (8)

**FIG. 1.**
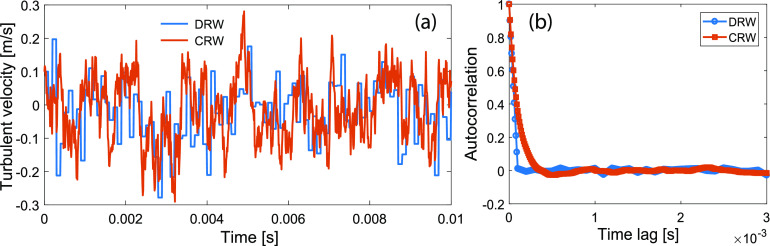
Comparison of DRW and CRW in predicting the evolution of turbulent velocities with parameters: $u_{0}^{\prime }$ = 0.1 m/s, *σ* = 0.1 m/s, Δ*t* = 10^−5^ s, *τ* = 10^−4^ s. (a) Turbulent velocity and (b) autocorrelation.

The advantage of CRW is its inherent nature of considering both successive velocity correlation and continuously stochastic behavior. This is a better representation of turbulence than DRW because DRW only considers the stochastic behavior discretely at each eddy interaction time scale and neglects the correlation of successive velocities. Figure 1(a) demonstrates an example of DRW vs CRW in predicting turbulent fluctuations ($u_{0}^{\prime }$ = 0.1 m/s, *σ* = 0.1 m/s, Δ*t* = 10^−5^ s, *τ* = 10^−4^ s). This example shows a continuously varying velocity signal with the behavior of a Markov chain (not a completely white noise signal) in CRW, compared to the discrete velocity signal in DRW. The plot of the autocorrelation function [Fig. 1(b)] shows that the velocity maintains its “memory” beyond the time scale of eddy interaction in CRW, whereas the velocity autocorrelation in DRW drops to zero after the eddy interaction time scale.

### C. Modeling of jet

Strong respiratory flows such as coughing and sneezing can be treated as buoyant jets due to their violent initial velocity and temperature difference between exhaled fluids and ambient air (Bourouiba *et al.*, 2014). For pure jets with no buoyancy, streamwise velocity is assumed to maintain its initial velocity within a short distance known as zone-of-flow-establishment (ZFE), typically 5–12 source diameter *D* (i.e., mouth opening in coughing and sneezing). In our model, we take ZFE = 6.2*D* for round jets (Lee and Chu, 2003). It is typically assumed that radial velocity is zero and no turbulence is present in the ZFE. Beyond the ZFE, the mean streamwise velocity decreases linearly with increasing distance away from the source within a well-defined flow region known as zone-of-established-flow (ZEF): (1) the streamwise velocity follows a Gaussian distribution in the cross section of the jet and (2) the radial velocity is much smaller in the jet, and its value at the jet boundary is known as entrainment velocity. The streamwise (*U*_*r*_) and radial (*V*_*r*_) velocities are written as (Lee and Chu, 2003; Wei and Li, 2015)

$$U_{r}=U_{c}\exp\left(-r^{2}/b_{g}^{2}\right),$$ (9)

$$V_{r}=\alpha_{j}U_{c}\frac{1-\exp\left(-r^{2}/b_{g}^{2}\right)-\left(\beta_{j}/\alpha_{j}\right)\left(r^{2}/b_{g}^{2}\right)\exp\left(-r^{2}/b_{g}^{2}\right)}{r/b_{g}},$$ (10)

where centerline velocity *U*_*c*_ = 6.2*U*_0_(*D*/*x*), jet entrainment coefficient *α*_*j*_ = 0.057, jet width growth rate *β*_*j*_ = 0.114 (Wei and Li, 2015), and *b*_*g*_ is the Gaussian jet half-width that defines the boundary of the jet.

**FIG. 2.**
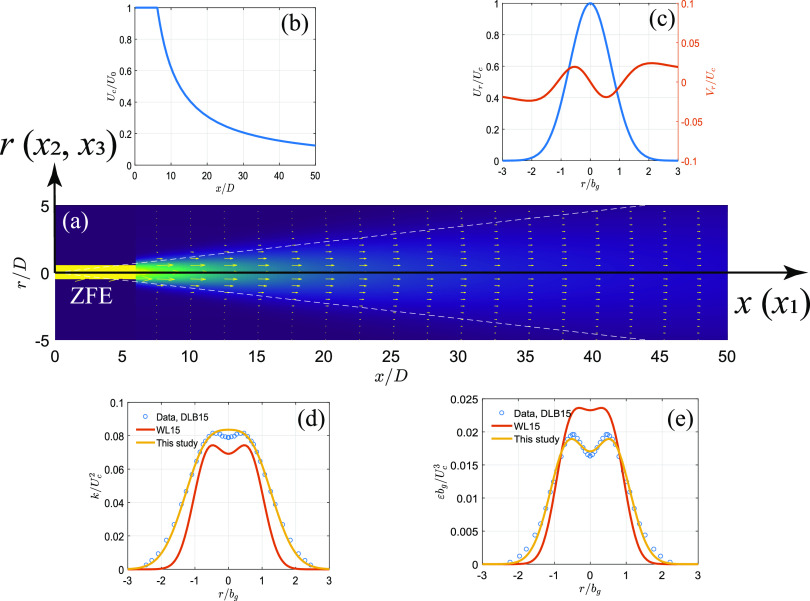
The schematic representation of the modeled jet: (a) velocity field and the coordinate system, (b) centerline velocity as a function of streamwise direction, (c) radial profile of streamwise and radial velocities, (d) radial profile of TKE, and (e) radial profile of the dissipation rate. Note that all parameters are plotted in normalized formats. DLB15 refers to the experimental data in Darisse *et al.* (2015), WL15 refers to the equations used in Wei and Li (2015).

Equations (9) and (10) define the mean flow field of the air jet in coughing and sneezing. In addition, turbulent statistics in the jet is needed to define necessary parameters for random walk [i.e., *α* and *β* in Eq. (7)]. In the model of Wei and Li (2015), the equations for rms velocity fluctuation and turbulent dissipation rate are fitted to the simulation data of a computational fluid dynamics (CFD) modeling of a sediment-laden jet in water (Chan *et al.*, 2014). In this paper, we use the experimental data from an air jet with a source Reynolds number of *Re*_*j*_ = *U*_0_*D*/*ν* = 1.4 × 10^5^ (Darisse *et al.*, 2015). Following a similar formula in Chan *et al.* (2014), we define the profile of turbulent kinetic energy and dissipation rate as follows:

$$k=U_{c}^{2}c_{1}\left[\exp\left(-c_{2}{\left(r/b_{g}-c_{3}\right)}^{2}\right)+\exp\left(-c_{2}{\left(r/b_{g}+c_{3}\right)}^{2}\right)\right],$$ (11)

$$\varepsilon =\left(U_{c}^{3}/b_{g}\right)c_{4}\left[\exp\left(-c_{5}{\left(r/b_{g}-c_{6}\right)}^{2}\right)+\exp\left(-c_{5}{\left(r/b_{g}+c_{6}\right)}^{2}\right)\right],$$ (12)

with fitted coefficients *c*_1_ = 0.0667, *c*_2_ = 1.079, *c*_3_ = 0.6853, *c*_4_ = 0.0178, *c*_5_ = 1.963, *c*_6_ = 0.6126 to the reported data in Darisse *et al.* (2015). A schematic presentation of the jet is given in Fig. 2, where all parameters in Eqs. (9)–(12) are plotted. Note that the radial profiles of turbulent fields (*k* and *ε*) are different from those in Chan *et al.* (2014) [used in Wei and Li (2015)].

### D. Model framework

The model is coded in open-source language Python with the scientific computing library “SciPy” and package “sdeint” for solving the ODE and SDE systems. The model solves for location (*x*_*i*_), velocity (*u*_*p*,*i*_), mass (*m*_*p*_), temperature (*T*_*p*_), and turbulent fluctuating velocity ($u_{i}^{\prime }$) in each time step. We apply a fifth order integrator using adaptive time steps with an absolute tolerance parameter of 10^−8^ to obtain the sub-micron accuracy. The model has several separated modules to compute mean flow velocity (*U*_*i*_) and properties of particles and air. We follow the equations in Kukkonen *et al.* (1989) (see their Appendix) to calculate the thermodynamic properties of the liquid droplet, vapor, and air. All droplet properties are updated every time step in the simulation. The mean flow and the turbulent statistics are calculated based on the initial condition given in the simulation. These parameters are used in the ODE and SDE solver until a model termination criterion (settle-on-ground or dry-out) is satisfied.

## III. MODEL VALIDATION

**FIG. 3.**
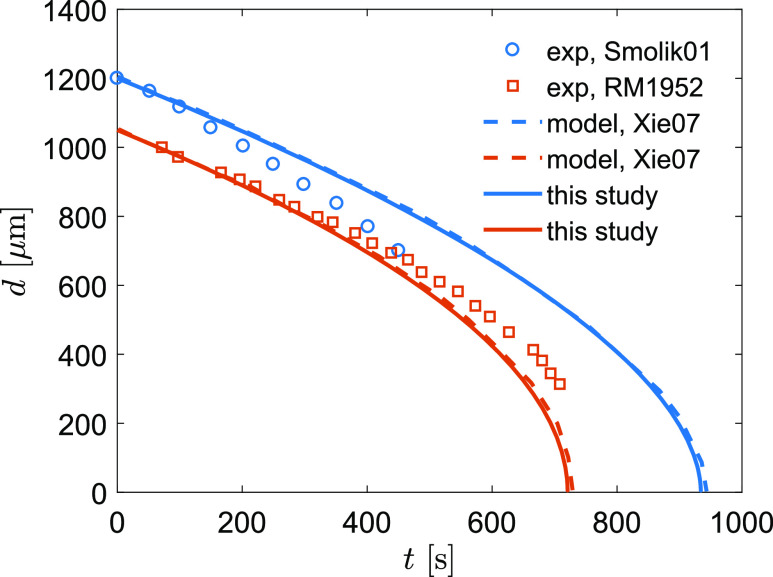
Comparison of droplet shrinkage between modeling and measurements. Results from the literature include Smolik01 [Smolik *et al.* (2001)], RM1952 [Ranz and Marshall (1952)], and Xie07 [Xie *et al.* (2007)].

The model is validated to measurement data and is compared with other modeling work in the literature. First, we validate the evaporation of a droplet in the condition of at-rest and uniform flow. We follow the modeling validation of Xie *et al.* (2007), who compared their modeling results with the measurements in Ranz and Marshall (1952) and Smolik *et al.* (2001). Ranz and Marshall (1952) measured the shrinkage of a water drop (*d*_*p*_ = 1.1 mm, *T*_*p*_ = 282 K) in dry air [*T*_*a*_ = 298 K, relative humidity (RH) = 0%]. Smolik *et al.* (2001) measured the shrinkage of a drop (*d*_*p*_ = 1.2 mm, *T*_*p*_ = 287 K) in a constant air stream (*U*_*a*_ = 0.203 m/s, *T*_*a*_ = 297 K, RH = 35%). The measurement-modeling comparison of droplet shrinkage as a function of time is shown in Fig. 3. Our modeling and Xie *et al.* (2007) show almost identical results because the same set of mass and heat transfer equations were used. The slight difference in model results is likely due to the numerical schemes. Xie *et al.* (2007) used fourth-order Runge–Kutta and we applied the fifth order SciPy ODE integrator based on the variable-coefficient ODE (VODE) with backward differentiation formulas (BDFs) for stiff problems. The comparison shows that the model results generally follow the data with an overestimate of shrinkage in the at-rest condition (*R*^2^ = 0.866) and underestimate of shrinkage in the uniform flow condition (*R*^2^ = 0.827). A considerable discrepancy between the model result and the data of Smolik *et al.* (2001) suggests the need for a better representation of the turbulent transfer of mass and heat in future studies.

To validate the model prediction of particle spreading in air flow, we compare our model results with the measurements of dispersion of four different inert particle types (see Table I) in a grid-generated turbulence field (Snyder and Lumley, 1971). The mean flow in the experiment is upward with a velocity of *W*_*a*_ = 6.5 m/s. The turbulent fluctuations were measured in the experiment and were used to derive the turbulent kinetic energy and dissipation rate (Bocksell and Loth, 2001),

$$k=\frac{W_{a}^{2}}{2}\left(\frac{1}{42.4\left(z/M-16\right)}+\frac{2}{39.4\left(z/M-12\right)}\right),$$ (13)

$$\varepsilon =\frac{W_{a}^{3}}{2M}\left(\frac{1}{42.4{\left(z/M-16\right)}^{2}}+\frac{2}{39.4{\left(z/M-12\right)}^{2}}\right),$$ (14)

where *z* is the vertical distance to the grid and *M* = 0.0254 m is the grid spacing.

**TABLE I.** Particle parameters in model validation of turbulent dispersion.

Particle type	Hollow glass	Corn pollen	Glass	Copper
Diameter (*μ*m)	46.5	87	87	46.5
Density (kg/m^3^)	260	1000	2500	8900
Terminal velocity (m/s)	0.017	0.194	0.439	0.479
Relaxation time (s)	0.002	0.020	0.045	0.049

**FIG. 4.**
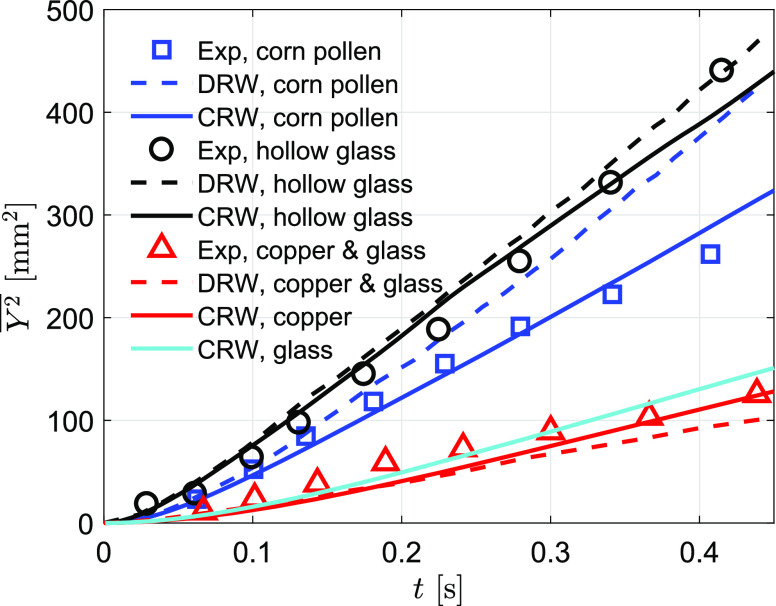
Comparison of particle spreading between modeling and measurements. Experimental data are from Snyder and Lumley (1971), and the DRW result is from Wei and Li (2015).

The measurement-modeling comparison of the particle spreading is shown in Fig. 4. Snyder and Lumley (1971) showed that the turbulent flow in the test region (68 < *z*/*M* < 168) is nearly isotropic and the spreading perpendicular to the mean flow direction is nearly homogeneous. The measurement data show that the lightest particles (hollow glass) spread about 450 mm^2^ and the heaviest particles (copper) spread about 120 mm^2^ within 0.4 s–0.5 s after the particles pass the first measurement station. Glass has a similar particle relaxation time as copper and hence has a similar spreading area due to similar particle–eddy interactions. The corn pollen is a median-weighted particle and has an intermediate spreading as a result of intermediate particle properties.

Wei and Li (2015) validated their cough jet model using this dataset with a DRW approach (shown as dashed lines in Fig. 4). They pointed out that the DRW model has difficulties in predicting median-weighted particles (corn pollen). Our comparison shows a general improvement of particle spreading simulation using CRW. The improvement is more profound for predicting median-weighted particles, corn pollen. The *R*^2^ value of model-measurement comparison for corn pollen increases from 0.49 (DRW) to 0.97 (CRW), an almost 100% improvement. The corn pollen tested in this experiment has the same density as the water droplet and has a diameter of 87 *μ*m, which is corresponding to the peak range of human respiratory flows (Han *et al.*, 2013). Hence, this improvement of modeling in this particle range is important in simulating particle dynamics in respiratory flows.

**FIG. 5.**
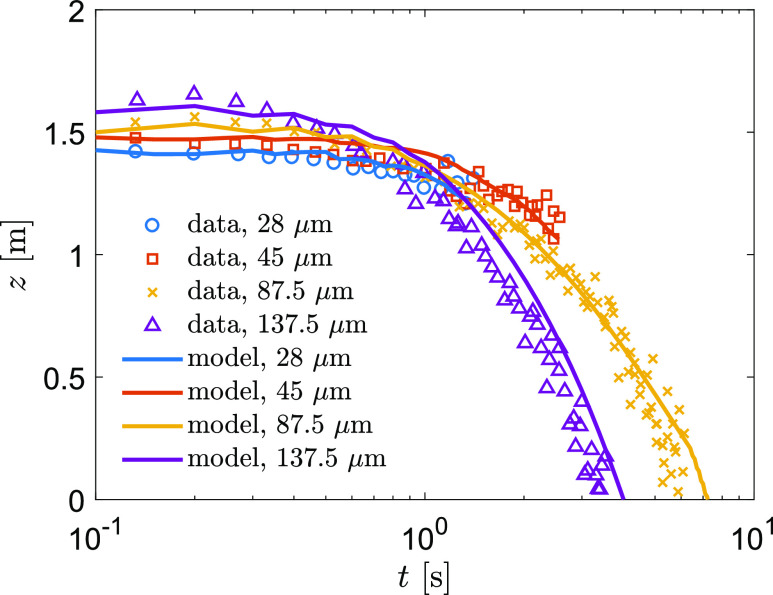
Comparison of particle altitude as a function of time in an upward jet.

Further model validation combines the droplet evaporation and location in a simulated cough jet flow. We simulate the experiment of Chao and Wan (2006)—an upward injection of droplets with a duration of 1 s in an environment of *T*_*a*_ = 21 °C and RH = 55%. The experimental data showed the droplets with diameters of 87.5 *μ*m and 137.5 *μ*m settled on the ground, whereas the droplets with diameters of 28 *μ*m and 45 *μ*m completely dried out in air (see Fig. 5). We initiated the model using the exact same condition of the experiment and tracked droplets for 10 s, following the measurement procedure (Chao and Wan, 2006). This validation demonstrates that our model predicted the airborne lifetime and the altitude of droplets well (Fig. 5).

## IV. MODEL APPLICATIONS

In this paper, we demonstrate a set of idealized scenarios and investigate the fundamental behavior of expiratory droplets in the environment. Although these idealized scenarios oversimplify the real-world conditions, however, the fundamental parameters depicted in these cases are crucial to understanding the virus transmission through various routes that are associated with these parameters (e.g., falling distance and airborne lifetime).

### A. Free-fall droplets

We first examine the airborne lifetime of a free-falling droplet. Here, we define airborne droplets as those remaining in air before they completely dry out or settle to the ground. We select droplets with diameters in the range of 1 *μ*m–1000 *μ*m, the primary range of droplet sizes in the human respiratory flows (Han *et al.*, 2013). Droplets smaller than 1 *μ*m evaporate quickly in typical indoor and outdoor environments (except for suppressed evaporation in extremely humid environments or condensation due to rapid droplet cooling). Xie *et al.* (2007) used 0.3 *μ*m as the criterion of nuclei formation, which is also used in this simulation.

This simulation is similar to what has been done in Xie *et al.* (2007), who showed settling or dry-out of a free-falling droplet up to 200 *μ*m (*T*_*p*_ = 33 °C) from 2 m with an ambient temperature of *T*_*a*_ = 18 °C. We adjust the model parameter to the initial height of 1.6 m, typical height of the human mouth, and the initial droplet temperature of 37 °C, typical body temperature of a healthy person. With these conditions, we examine the droplet properties in the ambient temperature range of 5 °C–35 °C (interval of 1 °C) and relative humidity of RH = 0%–95% (interval of 5%).

**FIG. 6.**
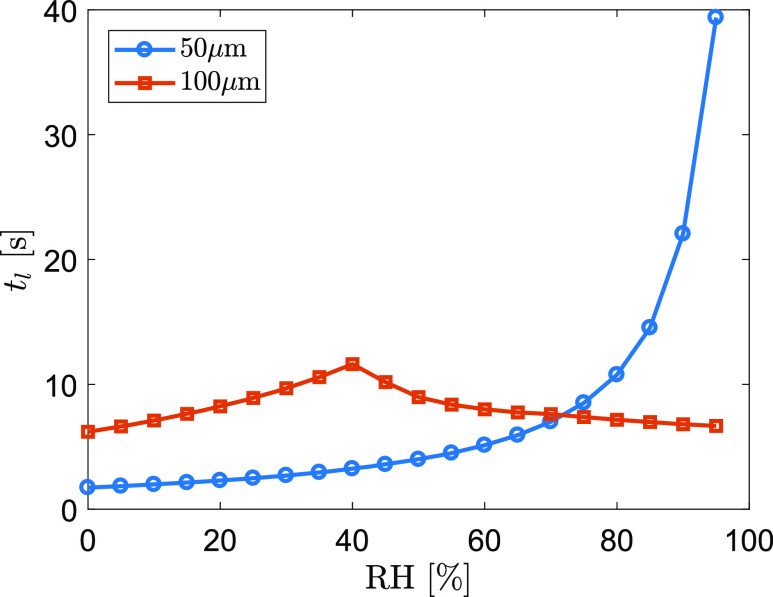
Airborne lifetime of a 50 *μ*m and a 100 *μ*m droplet as a function of RH at an ambient temperature of 22 °C.

Figure 6 shows an example of airborne lifetime of two droplets (*d*_*p*_ = 50 *μ*m and 100 *μ*m) as a function of relative humidity at an ambient temperature of 22 °C. For the 100 *μ*m droplet, the maximum lifetime of *t*_*l*_ = 11.6 s occurs at RH = 40% in the tested RH range. The maximum *t*_*l*_ corresponds to the coincident moment between the droplet settling on the ground and completely drying out. *t*_*l*_ is shorter in either less (dry-out before settled) or more (settled before dry-out) humid conditions. In contrast, a 50 *μ*m droplet does not have such a critical moment because it would completely evaporate in any of these tested relative humidity values. When air is completely dry (RH = 0%), the 50 *μ*m droplet evaporates within 1.7 s and the 100 *μ*m droplet evaporates within 6.2 s, slightly shorter than the model result of 2.0 s and 7.2 s, respectively, in Xie *et al.* (2007) in the condition of *T*_*a*_ = 18 °C and *z*_0_ = 2 m. It should be emphasized that the humidity can extend the airborne lifetime of a 50 *μ*m droplet by more than 23 times from RH = 0%–95%. This suggests that the airborne transport of median-sized droplets is extremely sensitive to the humidity.

**FIG. 7.**
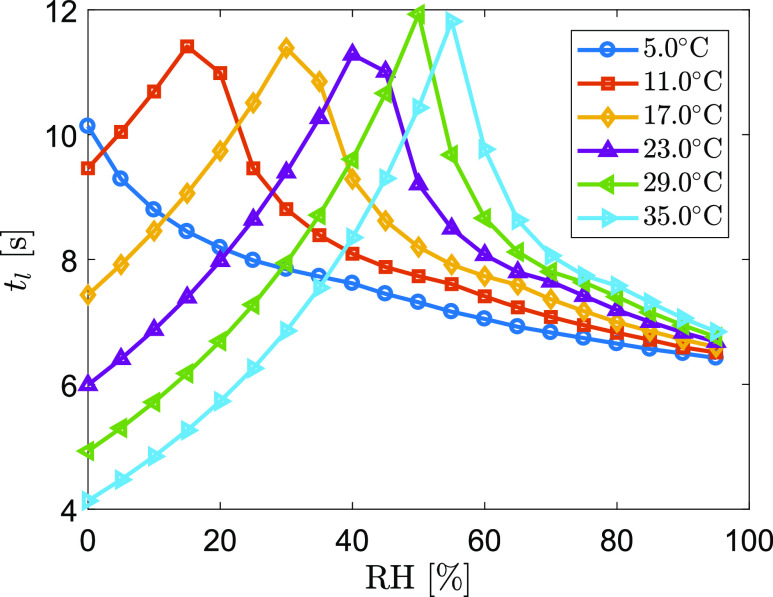
Airborne lifetime of a 100 *μ*m droplet as a function of RH at various ambient temperatures.

Ambient temperature also affects the lifetime of a droplet because the temperature strongly influences the thermodynamic properties of the droplets and associated vapor through heat exchange between droplets/vapor and the ambient air. Figure 7 shows an example of the lifetime curve of a 100 *μ*m droplet as a function of RH and temperature. This result shows how the maximum airborne lifetime shifts from humid toward dry air conditions when temperature decreases. In very humid air, decreasing temperature shortens the airborne lifetime due to slower evaporation if the droplet eventually settles on the ground. On the other hand, decreasing temperature in dry air would generally increase the airborne lifetime due to slower evaporation that slows down the dry-out process of the droplet. In most of indoor conditions, the combination of temperature and humidity lies in the middle range of the tested values, where the droplets with different sizes would have different and sensitive evaporation-settling dynamics. Hence, evaluating the interaction of individual expiratory droplets with the ambient environment is critical to understanding the fate and transport of virus-laden droplets.

**FIG. 8.**
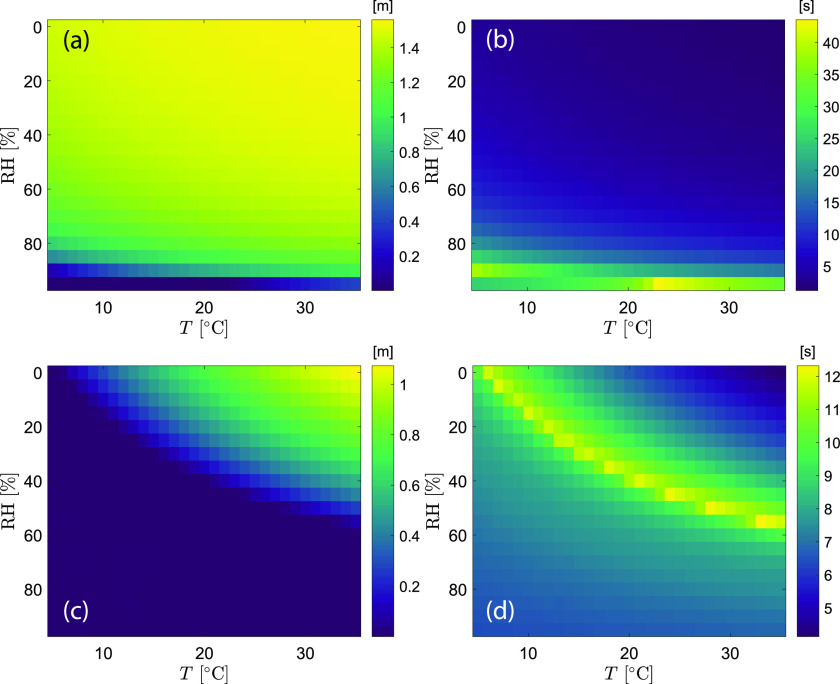
Map of the settling height and airborne lifetime as a function of temperature and humidity: (a) settling height, *d*_0_ = 50 *μ*m; (b) airborne lifetime, *d*_0_ = 50 *μ*m; (c) settling height, *d*_0_ = 100 *μ*m; and (d) airborne lifetime, *d*_0_ = 100 *μ*m. The grid size of the map is 1 °C and 5%.

The settling height of a droplet, defined as the final vertical location when dry-out or settle-to-ground occurs, is an intrinsic droplet property during evaporation in the free-falling condition. Similar to the airborne lifetime, it is a function of temperature and humidity for each droplet size. Figure 8 shows a data map of the settling height along with the lifetime for droplets of 50 *μ*m and 100 *μ*m. For the 50 *μ*m droplet, the settling height spans the range of 1.56 m (only 4 cm away from the initial height) to 0 (ground), with the lifetime in the range of 1.15 s–43.5 s. From the data map, a 50 *μ*m droplet is likely to evaporate completely before it falls to the ground under the free-fall condition in the majority of the indoor environments. The settling height of the droplet higher than 1 m (i.e., less than 0.6 m falling distance) occupies 87.4% of the total combinations of temperature and humidity. The temperature-humidity map for the 100 *μ*m droplet shows a clear boundary between settle-to-ground and dry-out. The longest and the shortest lifetime are 12.3 s and 4.1 s, respectively. The shortest lifetime corresponds to the fastest evaporation at a high temperature and dry air condition, resulting in the final settling height of 1.07 m, equivalent to 53 cm falling distance. The model result shows that a 100 *μ*m droplet would settle to the ground for 62.6% of the combinations of temperature and humidity in the tested range.

### B. Droplet dynamics in a “continuous” cough jet

We further apply the model to simulate an idealized case of human cough generated flows. Human cough can be treated as a momentum jet immediately after the flow being expelled. When the temperature of the expiratory fluid is higher than ambient air, a buoyancy driven plume effect would lift the flow upward, enhancing the upwelling of the droplets and leading to longer transport time in air (Bourouiba *et al.*, 2014).

To initialize the jet modeling, the exit velocity is a critical parameter that determines all mean and turbulent properties in the jet and the flow associated interactive terms such as turbulent enhanced mass and heat transfers. In this study, we use initial jet velocity *U*_0_ = 10 m/s, as the same value as used in the model of Wei and Li (2015). This velocity is close to the literature reported values in human coughing flows (Chao *et al.*, 2009; Kwon *et al.*, 2012). We apply the mouth opening diameter of 2 cm according to the measured mouth opening area in Gupta *et al.* (2009), which was later used in the modeling in Wei and Li (2015).

**FIG. 9.**
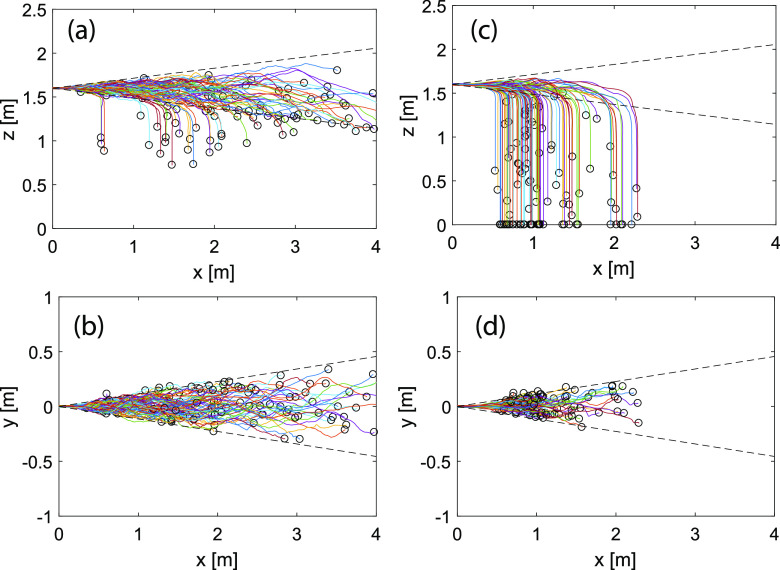
Droplet trajectories in cough jets: (a) 50 *μ*m, *x*–*z* plane; (b) 50 *μ*m, *x*–*y* plane; (c) 100 *μ*m, *x*–*z* plane; and (d) 100 *μ*m, *x*–*y* plane. *x*: streamwise direction, *y*: radial direction, and *z*: vertical direction. The dashed line shows the Gaussian velocity boundary defined as *b*_*g*_ = 0.114*x*. *T*_*∞*_ = 20 °C.

A key feature of our model is using CRW to better simulate turbulent velocities, which are critical to the spreading of the droplets in the near field of cough jets (Wei and Li, 2015). To demonstrate the performance of CRW in cough jets, we simulate an isothermal jet by releasing 100 droplets with the same diameter (two cases: 50 *μ*m and 100 *μ*m) one by one at a time interval of 0.1 s. We assume that there is no ambient flow and turn off the evaporation by defining RH = 100%. Droplet trajectories show that majority of the 50 *μ*m droplets are retained in the jet region and some droplets reach 4 m (see Fig. 9). In contrast, almost all 100 *μ*m droplets escape from the jet followed by a free-fall process. All droplets travel within 2.5 m in the streamwise direction.

From Fig. 9, we observe that most of the droplets are bounded within the Gaussian width of the jet (2*b*_*g*_ with *b*_*g*_ = 0.114*x*). Our result shows smaller spreading than that reported in Wei and Li (2015) using DRW. The boundary of particle trajectories in the DRW model tracks closer to the top-hat jet width 2*b*_*t*_ with $b_{t}=\sqrt{2}b_{g}$ (Wei and Li, 2015), which likely overestimates the droplet spreading since DRW may inaccurately capture the flow–particle interactions for expiratory droplets (see Fig. 4).

**FIG. 10.**
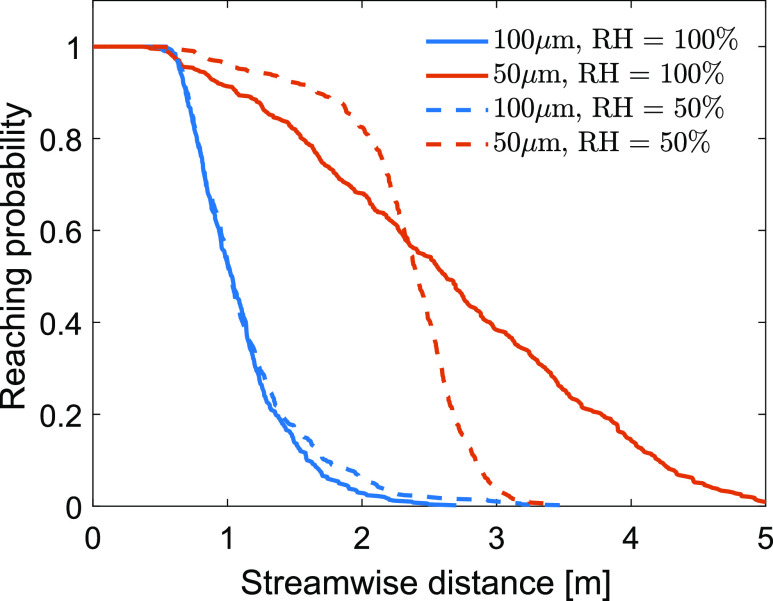
Reaching probability in the streamwise direction for 50 *μ*m and 100 *μ*m droplets at RH = 50% and 100%. *T*_*∞*_ = 20 °C.

To test the influence of relative humidity on the droplet spreading, we release 1000 equal-diameter droplets (two cases: 50 *μ*m and 100 *μ*m) and track their dispersion in the cough jet for 10 s at two relative humidity conditions (RH = 50% and 100%). This is similar to what has been simulated in the DRW model in Wei and Li (2015). We release droplets from 1.6 m above the ground, instead of 2 m as in Wei and Li (2015), and calculate the reaching probability in the streamwise distance (Fig. 10). Due to the strong initial velocity, all droplets reach 0.5 m (i.e., probability = 1). Some droplets settle to the ground within 1 m distance, leading to the decrease in reaching probability at further streamwise distances. At RH = 100%, all 100 *μ*m droplets settle to the ground within 2.7 m, whereas some 50 *μ*m droplets reach 5 m. Half population of the droplets reach 1.03 m and 2.62 m for 50 *μ*m and 100 *μ*m, respectively.

When RH = 50%, droplets shrink during the airborne travel, leading to the modification of the reaching probability curve. The model results show that the effect of relative humidity is profound for 50 *μ*m droplets but not as significant as for 100 *μ*m droplets. At RH = 50%, no droplets reach 3.5 m with the initial diameter of 50 *μ*m, reducing the maximum reaching distance by 34.6%. In the meantime, reducing RH also increases the likelihood of the droplet transport in the shorter region. For instance, the reaching probability at *x* = 2 m increases from 68.1% to 82.5% when RH decreases from 100% to 50%. This suggests that 50 *μ*m droplets tend to accumulate within a narrower range in the streamwise direction when RH decreases to 50%. At RH = 100%, the middle 60% of the droplets are within the 1.56 m–3.7 m range, and this range becomes 2.05 m–2.66 m at RH = 50%.

Different from the decrease in the maximum reaching distance for 50 *μ*m droplets, decreasing RH to 50% increases the maximum reaching distance by 29.6% for 100 *μ*m, from 2.7 m to 3.5 m. For the shorter regime (within 1 m), the probability curve almost does not respond to the change of RH from 100% to 50%, giving only 50% of the droplets reaching 1 m. The sensitivity of droplet transport in cough jets between different diameters in response to the RH changes varies significantly due to the non-linearly correlated dynamics in the processes of mass and heat transfer and the kinematics of droplets.

### C. Droplet dynamics in a “pulsating” cough jet

In a single coughing event, the cough jet is more like a pulsating flow with strong initial velocity, which rapidly decays to the normal breath condition. The duration of pulsating cough flow is typically 0.5 s (Gupta *et al.*, 2009). Wei and Li (2017) conducted an experiment by releasing particle-laden jets in water to simulate the cough jet using a constant velocity, a sinusoidal profile, and a profile of the gamma distribution function in a duration of 2 s. Here, we simulate a similar pulsating cough jet to track the particle trajectory and its shrinkage, using a constant exit velocity *U*_0_ = 10 m/s for 1 s. After 1 s, we stop the jet and let particles to settle in three different conditions: (1) no flow, (2) an upward flow with *W* = 0.1 m/s, and (3) a downward flow with *W* = −0.1 m/s. To simplify the physical process of droplets after the cough, we did not consider the influence of ambient turbulence, i.e., the droplets would be only advected by the ambient flow with their velocities determined at the end of the cough.

**FIG. 11.**
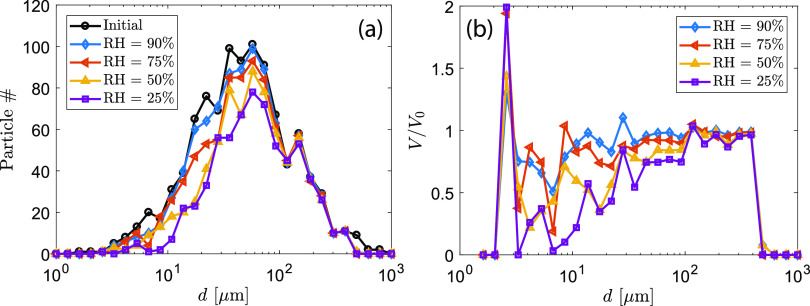
(a) Histogram plot of simulated droplets after 1 s cough. (b) Ratio of final volume to initial volume in each size bin before and after 1 s cough. Four different relative humidity values were simulated. *T*_*∞*_ = 20 °C.

The model takes a log-normal distribution of droplet diameters within the range of 1 *μ*m–1000 *μ*m and randomly draws 1000 samples from the distribution [see “initial” in Fig. 11(a)]. The modeled droplet size distribution is similar to the measured distribution in real coughs (Xie *et al.*, 2009). Our modeled data have a median droplet diameter of 51.3 *μ*m, a mean diameter of 85.9 *μ*m, and a standard derivation of 111.3 *μ*m. The evolution of the droplet locations and sizes at four tested humidity conditions is provided in the supplementary material.

First, we examine the total population changes of droplets that remain airborne after 1 s cough. Figure 11(a) shows the histogram plot of droplets that neither settle on the ground nor completely dry out. Large-sized droplets settle to the ground for all RH conditions. The number of droplets in the diameter range of 10 *μ*m–100 *μ*m decreases more dramatically with decreasing RH than other size ranges. This suggests that this size range is mostly sensitive to humidity. This result is also shown in the plot of the final-to-initial volume ratio in each droplet size bin [Fig. 11(b)].

**FIG. 12.**
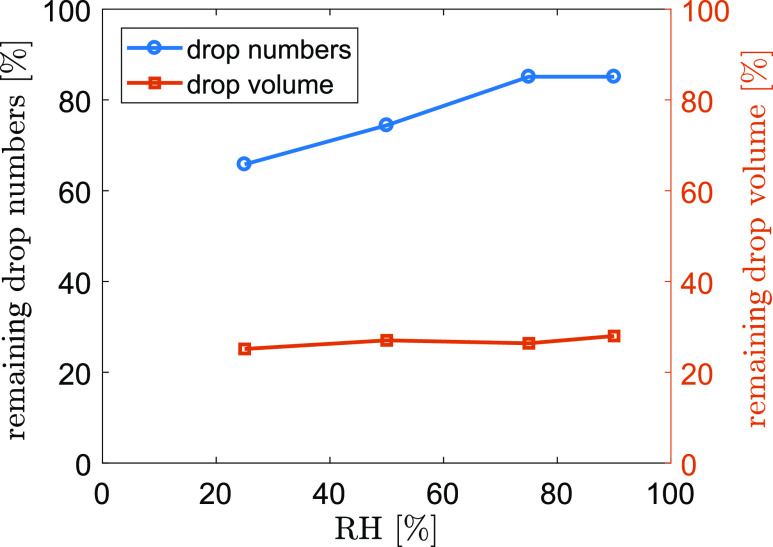
Percentage of numbers and volume of airborne droplets compared to the initial condition after 1 s cough. *T*_*∞*_ = 20 °C.

Figure 12 plots the percentage of numbers and volume of airborne droplets compared to the initial condition after 1 s cough at different RH conditions. The model results show that about 85.1% of the droplets remain airborne after 1 s at RH = 90%. 14.9% of the droplets either settle to the ground (2.6%) or completely evaporate (12.3%) within 1 s. When RH decreases, more droplets evaporate completely within 1 s, resulting in a significant decrease of airborne droplets (74.4% and 65.8% for RH = 50% and 25%, respectively). Despite the dramatic change in airborne droplet numbers, the total volume of airborne droplets does not change drastically due to the weak contribution to the volume from small droplets. The airborne droplets only occupy about 30% of the initial volume, which remains almost unchanged during 1 s cough throughout the tested humidity range. This suggests that the majority of the volume is contributed by those droplets that settle to the ground during the cough. For instance, at RH = 90%, 82.5% of the dry-out droplets only occupy 0.0004% of the total volume. If the virus load associated with the droplets is proportional to the volume, almost 70% of the virus would deposit on the ground during the cough. Hence, maintaining physical distance would significantly remediate the spreading of respiratory infectious diseases through reducing direct deposition of droplets onto people within short distances and through reducing the probability of inhalation of high concentration aerosols near the source.

**FIG. 13.**
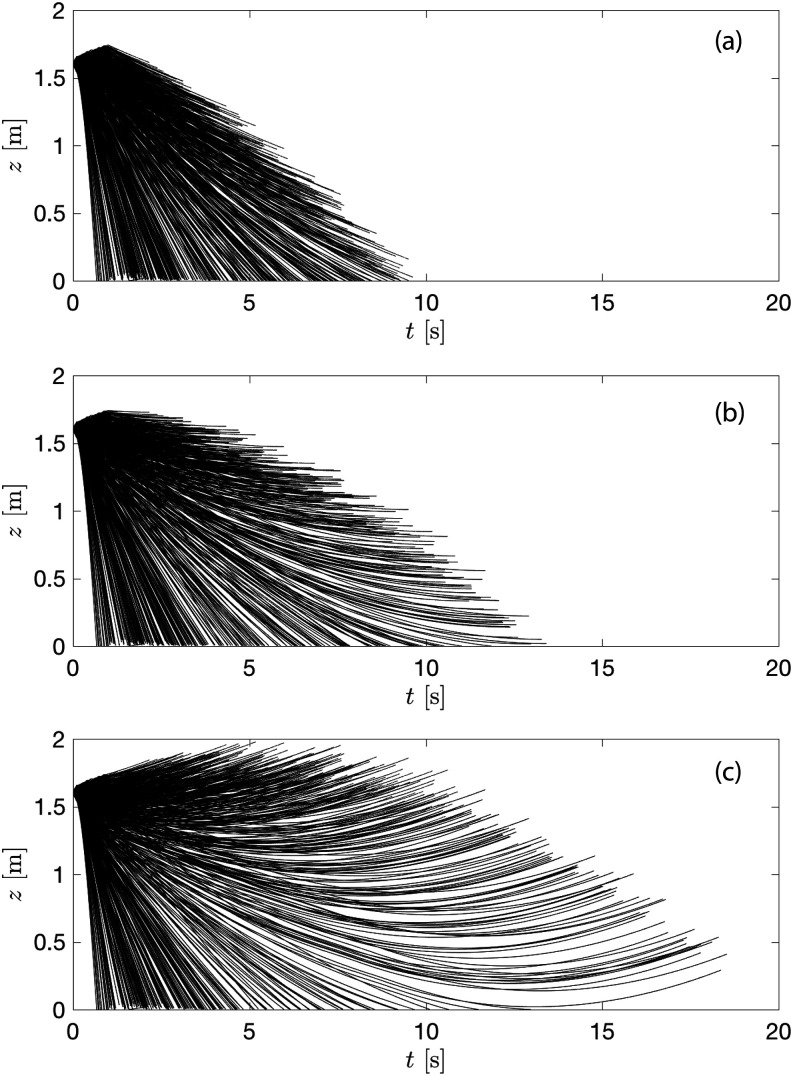
Height of the droplets as a function of lifetime under (a) downward flow, (b) no flow, and (c) upward flow at RH = 50% and *T*_*∞*_ = 20 °C.

When coughing occurs in the environment with ambient flows, the droplet airborne time and the droplet transport will be modified by additional dispersion depending on the strength of the mean and turbulent velocities. Figure 13 illustrates the modification of droplet airborne time by the mean flow. Here, we calculate the droplet vertical location as a function of time under downward flow, no flow, and upward flow conditions. The process is simplified by not accounting for turbulent fluctuations in ambient flows, assuming no horizontal velocities, and only applying the ambient flows after the completion of the cough. Note that such idealized flows do not exist in real indoor and outdoor environments but provide valuable quantitative information about how the dynamics of coughing droplets can be modified by simple advection of mean velocities.

**FIG. 14.**
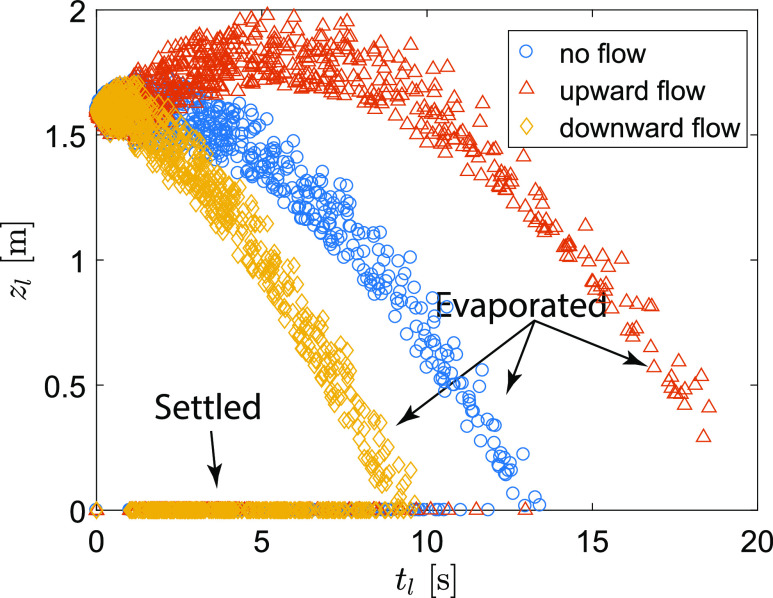
The relationship between the final height of each droplet and the correspondent final time for three different flow conditions at RH = 50% and *T*_*∞*_ = 20 °C.

The model results show that the longest droplet airborne time is 13.4 s with no ambient flow. This duration would be reduced by 28% (9.6 s) with the downward velocity of 0.1 m/s and increased by 38% (18.5 s) with the upward velocity of 0.1 m/s, respectively. Because the droplets undergo a continuous shrinkage during their transport in air, the modification of their airborne time would change their temporal and spatial distributions and hence would affect the transmission of respiratory diseases if the droplets carry infectious viruses. Figure 14 shows the final vertical locations of each droplet (*z*_*l*_) as the function of their fate (i.e., the maximal airborne lifetime, *t*_*l*_). When there is no ambient flow, 25.0% of the droplets settle before they completely evaporate in the air (occupying 72.6% of the initial total volume). In another word, 75% of the droplet population completely evaporate while airborne, accounting for 27.4% of the initial total volume. The percentage of settled droplets is modified by the ambient flow with a decrease of 5.7% and an increase of 7.1% in the upward (19.3% in population, occupying 72.2% of the initial total volume) and downward (32.1% in population, occupying 73.3% of the initial total volume) flow conditions, respectively. When the droplets are associated with infectious viruses, the settled droplets could result in fomite transmission and the completely evaporated droplets may cause airborne transmission of the respiratory infectious diseases through their nuclei that are further transported by air flows.

The airborne time, maximal reaching height, and percentage of airborne and settled droplets can be modified by the ambient conditions such as temperature and humidity. Table II summarizes the calculation for four different relative humidity and three flow conditions. Taking two extreme conditions (RH = 25% and 90%), the maximal airborne time can be extended in humid air by 194%, 343%, and 77% under no flow, upward flow, and downward flow, respectively. The change in maximal reaching height is only noticeable in the upward flow condition from 1.87 m to 3.2 m when changing RH from 25% to 90%. The droplets that reach high are those having small to median sizes because they can be easily carried by background flows and concurrently have adequate lifetime. Rising RH from 25% to 90% approximately doubles the percentage of settled droplets in all flow conditions. The droplets that settle to the ground are large ones, which contribute to the majority of the total volume. Even under the upward flow and the driest condition, the settled droplets occupy 97.8% of the total volume.

**TABLE II.** The parameters of settled and airborne droplets at different relative humidity and flow conditions. Only the percentage of settled droplets is shown, and the correspondent airborne percentage can be calculated by subtracting it from 100%.

	No flow				Upward flow, 0.1 m/s				Downward flow, 0.1 m/s			
Relative humidity (%)	25	50	75	90	25	50	75	90	25	50	75	90
Maximal airborne time (s)	10.8	13.4	19.6	31.8	13.7	18.5	30.6	60.7	8.3	9.6	11.6	14.7
Maximal height (m)	1.76	1.73	1.73	1.74	1.87	1.98	2.29	3.20	1.73	1.71	1.71	1.72
Percentage of settled droplets in population (%)	22.2	26.2	34.1	44.7	18.4	20.4	24.5	30.4	27.2	33.2	45.8	61.7
Percentage of settled droplets in volume (%)	98.5	98.9	99.4	99.7	97.8	98.2	98.7	99.2	99.0	99.4	99.8	99.9

## V. MODEL LIMITATIONS

Several assumptions or simplification is used in the current model and study. First, in violent expiratory events, droplets are generated from the source and then undergo a continuous breakup/coalescence process under strong shear. The non-Newtonian nature of droplets makes the droplet cascade a complex process and further affects the final droplet sizes in the downstream of the expiratory flows (Scharfman *et al.*, 2016). In the current model, we assume that the initial droplet size distribution is the final converged distribution and neglect the particle–particle interactions. Second, the flow in human respiratory events is puff and involves time-varying velocity at the source. The present study simplifies the flow as a jet and applies a constant initial velocity in the modeled cough event. The adjustment can be made by employing a time-varying ambient flow field and adding the buoyancy effect due to the temperature difference between the exhaled fluid and ambient air. Finally, the evaporation of droplets near the source would induce a localized humidity increase, which could potentially delay the evaporation of the droplets. Neglecting this effect could overestimate the droplet shrinkage, particularly for the high concentration, small size range droplets. These model limitations and caveats to the study are subject to future investigations.

## VI. CONCLUSION AND OUTLOOK

In this paper, we present a numerical model to quantify the dynamics of droplets from human expiratory events that are related to the transmission of respiratory infectious diseases. We apply a CRW model to improve the current shortcomings in predicting the spreading of median-sized droplets in DRW models. Our validation data show a nearly 100% improvement in predicting the spreading of droplets with environment-sensitive sizes.

We show the behavior of free-falling droplets in different temperature and humidity environments. A 50 *μ*m droplet has a large range of airborne lifetime (1.15 s–43.5 s) and settling heights (0 m–1.56 m), which originated at 1.6 m height at the temperature of 5 °C–35 °C and relative humidity of RH = 0%–95%. In the same combination of temperature and humidity, a 100 *μ*m droplet has a much narrower lifetime range (4.1 s–12.3 s), resulting in a narrower range of settling heights (0 m–1.07 m).

Simulating a continuous cough jet further illustrates the sensitivity of median-sized droplets to the ambient environment. Under a steady-state cough jet, the reaching probability of 100 *μ*m droplets in the streamwise direction does not change significantly when reducing relative humidity from 100% to 50%. The reaching probability of 50 *μ*m droplets is reshaped by the humidity, leading to a narrowed range in the streamwise direction as a result of enhanced evaporation.

Taking a realistic droplet size distribution and the duration of a cough jet, we show that 14.9% of the droplet population either settles to the ground (2.6%) or completely evaporates (12.3%) at RH = 90% within the coughing event (1 s). 85.1% of the droplet population remains in air and will be further transported by ambient flows. This remaining percentage decreases to 74.4% and 65.8% for RH = 50% and 25%, respectively. However, they only occupy about 30% of the total volume with negligible variations in the tested humidity range (RH = 25%–90%). Ambient flow further changes the transport and dynamics through modifying the settling, airborne travel, and dry-out of the droplets. Different combinations of ambient environments and flows result in different droplet airborne time and transport distances, as well as different percentages of droplets that could be the sources of airborne or fomite transmissions. In the tested parameter range, the droplets occupying the majority of the initial volume would eventually settle on the ground rather than completely evaporating in the air.

The model we developed from this study will better our understanding of the mechanisms for airborne pathogens, e.g., influenza. Previous studies suggested that influenza viruses are present in both fine and coarse aerosol particles exhaled by influenza patients, including those with sizes <5 *μ*m or >5 *μ*m (Milton *et al.*, 2013; Gralton *et al.*, 2013; Lednicky and Loeb, 2013; and Lindsley *et al.*, 2015). These studies suggested variation of particle sizes in influenza carrying particles; however, none of these studies have determined roles of particle sizes more than 50 *μ*m (Milton *et al.*, 2013). Thus, this study highlighted the importance of evaluating the transport and fate of various sizes of droplets in aerosol transmission of airborne pathogens, such as influenza. As a future study, we plan to apply the model to study both fine and coarse droplets in influenza transmission.

## SUPPLEMENTARY MATERIAL

The supplementary material videos show the transport process of droplets in 1 s cough at four humidity conditions. The supplementary file shows the final locations of the droplets and their sizes after 1 s cough.

## DATA AVAILABILITY

The data that support the findings of this study are available from the corresponding author upon reasonable request.
